# The Value of Expression of M2-PK and VEGF in Patients with Advanced Gastric Cancer

**DOI:** 10.1007/s12013-013-9601-0

**Published:** 2013-04-30

**Authors:** Lanning Yin, Xiang Wang, Changjiang Luo, Haipeng Liu, Ling Zhang, Hong Zhang, Youcheng Zhang

**Affiliations:** 1 Department of General Surgery, Lanzhou University Second Hospital, 82 Cuiyingmen, Chengguan District, Lanzhou, 730030 Gansu Province China; 2 Department of Gastroenterology, Lanzhou University Second Hospital, Lanzhou, 730030 Gansu Province China

**Keywords:** M2 pyruvate kinase isoenzyme, Vascular endothelial growth factor, Advanced gastric cancer

## Abstract

Glycolytic pyruvate kinase isoenzyme type M2 (M2-PK) plays a key role in tumor metabolism and energy production. Vascular endothelial growth factor (VEGF) is critical in regulating angiogenesis which is an essential process required for tumor growth and metastasis. These two genes may function in accordance with tumor development. The purpose of this study was to investigate the relationship between the expression of M2-PK and VEGF, and their association with clinicopathological features in patients with advanced gastric cancer. Expression of M2-PK and VEGF were examined in 142 cases of paraffin-embedded tissue blocks from patients with advanced gastric cancer. M2-PK expression was found to strongly correlate with that of VEGF (*r* = 0.718). In addition, expression of M2-PK and VEGF correlates with tumor size (*p* = 0.0001, and *p* = 0.0017, respectively), depth of invasion (*p* = 0.0024, and *p* = 0.0261, respectively), and lymph node metastasis (*p* = 0.036, and *p* = 0.028, respectively). The high expression levels of M2-PK and VEGF may indicate poor prognosis in patients with advanced gastric cancer.

## Introduction

Gastric cancer is a significant global health burden. Approximately 934,000 new cases of gastric cancer are diagnosed annually (representing 8.6 % of all new cancer cases) [1]. Nearly two-thirds of all gastric cancer cases are found in developing countries, with 42 % in China alone [2]. Gastric cancer remains an aggressive disease with a high mortality rate. Despite a marked decrease in the mortality of gastric cancer in most areas of the world [3, 4], this malignancy remains the second leading cause of cancer-related death worldwide. It has a 5-year survival rate of ~20 % [5–7]. Post-operative recurrence is a major problem, and is often the ultimate cause of death. The reported major factors determining the prognosis include depth of tumor invasion, lymph node metastasis, and tumor size [8].

Tumor angiogenesis plays a critical role in tumor growth and metastasis [9–11]. Any increase in a tumor mass must be preceded by an increase in the microvasculature to deliver nutrients and oxygen to the tumor and remove products of tumor metabolism. Without new blood vessels, most tumors would never grow beyond 1–2 mm in diameter and would remain localized to the primary site [10].

Tumor cells generally display high rates of aerobic glycolysis [11]. The glycolytic pyruvate kinase isoenzyme type M2 (M2-PK) plays a key role by channeling glucose carbons either into synthetic processes or toward glycolytic energy production. In tumor cells, M2-PK is predominantly present as a dimeric form known as tumor M2-PK. Dimerization M2-PK appears to be caused by direct interaction between M2-PK and certain oncoproteins. This is thought to be a regulatory mechanism which allows tumor cells to survive in environments with varying oxygen and nutrient supplies [12].

In this study, we aimed to examine the expression of M2-PK and VEGF and determine whether these biological parameters could be used to predict the outcome of patients with advanced gastric cancer.

## Materials and Methods

### Patients and Tumor Specimens

We collected 142 paraffin-embedded tissue blocks from patients with advanced gastric cancer who underwent curative surgery at Lanzhou University Second Hospital between January 2005 and December 2007. None of these patients received chemotherapy or radiation therapy before surgery. However, all patients had received six cycles of standard post-operative adjuvant chemotherapy with 5-fluorouracil (5-FU) and leucovorin (LV) plus oxaliplatin. Patients were regularly followed-up at the outpatient clinic after surgery and the survival data as of January 2011 were obtained through hospital records. The median follow-up duration was 32 months (range: 1–66 months). The three-year survival rate is 35.9 %. Forty-one patients died of cancer recurrence within one year, and 75 patients died within two year of surgery. Fifty-one and 31 patients were disease free at three and five years post-surgery, respectively.

### Clinicopathological Data

Age at surgery, gender, tumor factor, tumor invasion, tumor size, lymph node metastasis, tumor stage, Borrmann type, and histologic grading were recorded in the survivors. Stage classification was according to the Union for International Cancer Control (UICC) system [13].

### Immunohistochemical Staining

Paraffin-embedded tissues were cut into 4 μm sections, deparaffinized with xylene and washed with PBS. After blocking with 1 % goat serum in PBS for 15 min, slides were incubated with a polyclonal rabbit anti-VEGF (Santa Cruz, CA, USA) (dilution: 1:100), or a polyclonal rabbit anti-M2-PK (ScheBo Biotech, Giessen, Germany) (dilution: 1:100) for 45 min at room temperature. The anti-M2-PK only recognizes the dimeric form of M2-PK, which is the predominant form of M2-PK in tumor tissues [14]. The slides were washed with PBS and incubated with the appropriate biotinylated secondary antibodies. The slides were then washed and incubated with streptavidin–peroxidase (DAKO, Shanghai, China) according to the manufacturer’s instructions, followed by incubation with 3,3′-diaminobenzidine (DAB) (DAKO, Shanghai, China) and counterstained with hematoxylin. The stained slides were examined by two pathologists who were blinded to the clinical information and the nature of specimens. The immunoreactivity was scored as shown in Table 1.

**Table 1:** The scoring system for immunohistochemistry

Staining	Score
No	0
Positive in <5 % of tumor cells	1
Positive in 5–25 % of tumor cells	2
Positive in >25 % of tumor cells	3

### Statistical Analysis

The statistical analysis was performed using SPSS (version 12.0). Correlation of M2-PK and VEGF staining with clinicopathological parameters was analyzed using Chi square test. Correlation between M2-PK and VEGF was determined by the Pearson Correlation Coefficient analysis. Kaplan–Meier analysis was used to assess the patient survival. A *p* value of <0.05 was considered statistically significant.

## Results

### Clinicopathological Findings

As summarized in Table 2, gastric cancer is more common in men than in women. Most gastric cancer patients are younger than 60 years when diagnosed. Most of these gastric cancer patients (89/142, 62.7 %) had T2 tumors, followed by T3 (35/142, 24.6 %), and T4 tumors (11/142, 7.7 %), whereas only a small number of patients were in T1 phase (7/142, 5 %). When the tumors were stratified according to the extent of invasion into early (T1) and late (T2–T4) stages, it was revealed that in the vast majority of patients (135/142, 95 %), the tumors had reached late stages, with most having large tumors (defined as >3 cm, 96/142, 67.6 %) and lymph node metastasis (125/142, 88 %). Using either UICC or Borrmann staging system, it was revealed that the majority of patients were in stages II and III (75.4 and 88.7 %, respectively). Although these tumors were diagnosed at relatively late stages, they generally showed either moderate (84/142, 59.2 %) or well (31/142, 21.8 %) differentiation, with only 19 % were poorly differentiated.

**Table 2:** Clinicopathological findings in 142 patients with gastric cancer

Variables	Number of cases (%)
Sex	
Female	45 (31.7)
Male	97 (68.3)
Age (years)	
<60	81 (57.0)
>60	61 (43.0)
Tumor invasion	
T1	7 (5.0)
T2	89 (62.7)
T3	35 (24.6)
T4	11 (7.7)
Tumor size	
<3 cm	46 (32.4)
3–5 cm	69 (48.6)
>5 cm	27 (19.0)
Lymph node metastasis	
N0	17 (12.0)
N1	28 (19.7)
N2 and/or N3	97 (68.3)
UICC stage	
I	21 (14.8)
II	44 (31.0)
III	63 (44.4)
IV	14 (9.8)
Borrmann stage	
I	5 (3.5)
II	62 (43.7)
III	64 (45.1)
IV	11 (7.7)
Differentiation status	
Well	31 (21.8)
Moderate	84 (59.2)
Poor	27 (19.0)

### Immunohistochemical Features

**Fig. 1:**
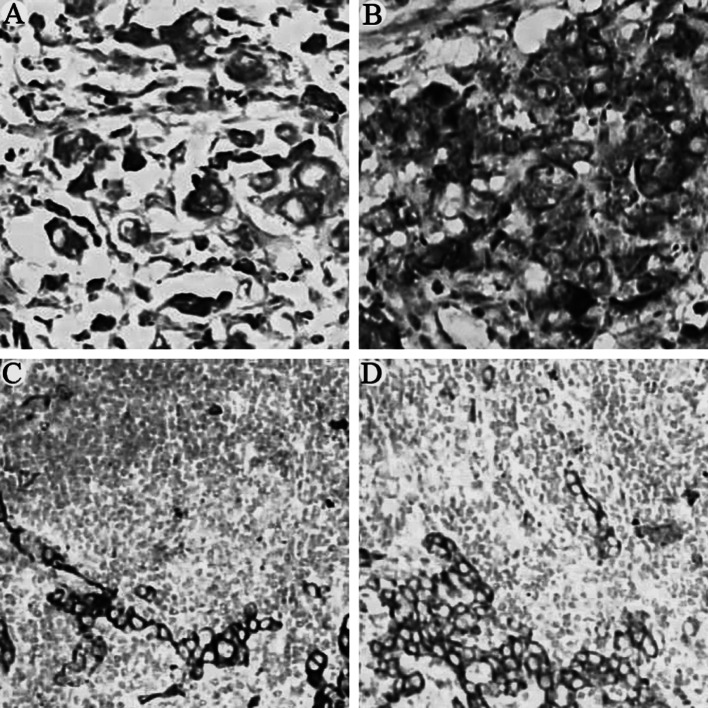
Expression of M2-PK (**a**, ×200) and VEGF (**b**, ×200) in primary gastric cancer tissues and gastric cancer metastasized to lymph nodes (**c** and **d**, respectively, all ×100)

Positive staining for M2-PK and VEGF was observed in most cases of gastric cancer tissues (93/142, 65.49 %, and 87/142, 61.27 %, respectively). Both M2-PK and VEGF are mainly expressed in the cytoplasm or on the membrane of the cancer cells. Table 3 summarizes the detailed immunohistochemical staining data and their correlation with the clinicopathological features. Typical immunostaining results were shown in Fig. 1.

**Table 3:** Expression of M2PK and VEGF, and their correlation with clinicopathological features

Variables	M2PK scores		*p* value	VEGF scores		*p* value
	0–1	2–3		0–1	2–3	
Gender						
Male	19	79		14	84	
Female	13	32	0.16	12	33	0.62
Age						
<60	21	59		15	65	
≥60	10	52	0.85	11	51	0.06
Tumor size						
<3 cm	13	33		24	22	
3–5 cm	15	54		13	56	
>5 cm	2	26	0.0001*	3	26	0.0017*
Tumor invasion						
T1	7	0		11	14	
T2	24	66		28	42	
T3	12	20		12	23	
T4	4	8	0.0024*	3	9	0.0261*
UICC stage						
I	7	15		6	12	
II	13	27		18	35	
III	19	38		19	38	
IV	6	11	0.068	5	7	0.122
Lymph node metastasis						
No	7	11		6	12	
Yes	44	81	0.036*	38	86	0.028*
Histologic grade						
Well	2	28		4	26	
Moderate	22	64		17	69	
Poor	6	20	0.909	3	23	0.123
Borrmann						
I	1	4		0	5	
II	15	47		11	51	
III	13	51		13	51	
IV	2	9	0.579	1	0	0.926

* *p* < 0.05 was considered statistically significant

### Correlation Between the Expression of M2-PK and VEGF, and the Clinicopathological Features

M2-PK and VEGF were significantly correlated with tumor size (*p* = 0.0001 and *p* = 0.0017, respectively), depth of invasion (*p* = 0.0024 and *p* = 0.0261, respectively), and lymph node metastasis (*p* = 0.036 and *p* = 0.028, respectively). The expression of both proteins did not correlate with gender, differentiation status, and tumor staging either by Borrmann classification or UICC system. These data were summarized in Table 3.

### Correlation Between the Expression of M2-PK and VEGF, and Patient Survival

**Fig. 2:**
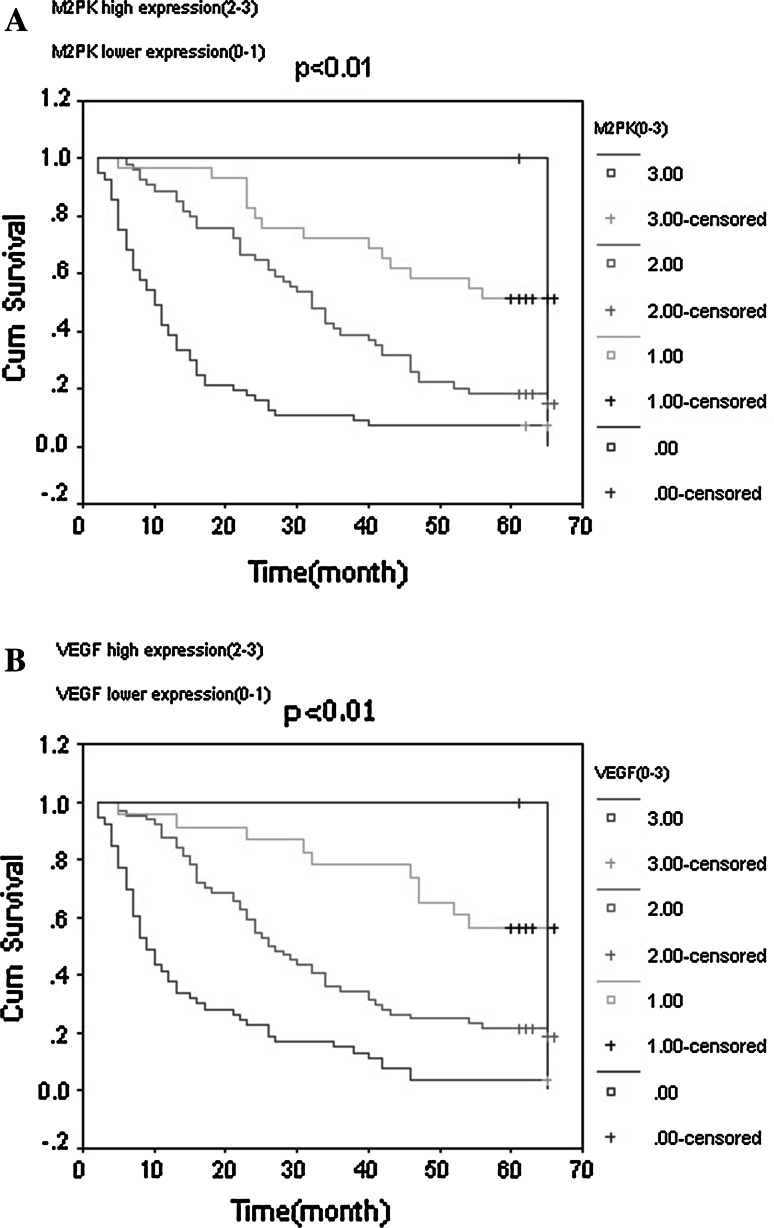
Patients expressing lower levels of M2-PK (**a**) and VEGF (**b**) show significantly better 5-year overall survival compared to those with higher expression levels of both protein (*p* < 0.01)

The prognostic value of M2-PK and VEGF on patients with advanced gastric cancer was evaluated and compared between patients with high and low expressions of both proteins. Using a Kaplan–Meier curve, we found that low M2-PK and VEGF expression in tumor tissue was an independent predictor for poor prognosis in patients with advanced gastric cancer. The five-year overall survival rate in patients expressing lower levels of M2-PK and VEGF was significantly better than those expressing higher levels of both proteins (*p* < 0.01) (Fig. 2a, b, respectively).

### Univariate and Multivariate Analysis of Prognosis Variables

To identify the variables of potential prognostic significance in patients with advanced gastric cancer, univariate and multivariate analyses were carried out using the Cox proportional hazard model to compare the impact of the expression levels of M2-PK and VEGF and other clinical pathological parameters on the prognosis. It has been revealed that M2-PK and VEGF expression, tumor size and UICC stage were significant prognostic factors in these patients (Table 4).

**Table 4:** The mean survival time of the patients in different groups of various prognostic factors

Risk factors	Mean ± SEM^a^	*p* value^b^
Gender		
Female	30.90 ± 22.55	NS
Male		29.40 ± 21.47
Age (years)		
<60	33.95 ± 22.73	NS
≥60	26.60 ± 19.31	
M2PK expression		
0–1	48.90 ± 18.14	<0.0001
2–3	24.55 ± 19.63	
VEGF expression		
0–1	51.28 ± 17.16	<0.01
2–3	35.29 ± 19.84	
Tumor size		
<3 cm	33.24 ± 21.19	
3–5 cm	31.33 ± 22.35	NS
≥5 cm	20.82 ± 19.19	0.017
Tumor invasion		
T1–T2	32.72 ± 22.80	
T3–T4	26.83 ± 17.19	0.061
Lymph node metastasis		
N0–N1	31.47 ± 18.63	
N2 or N3	27.45 ± 22.25	NS
UICC stage		
I–II	43.17 ± 21.00	
III–IV	24.11 ± 22.57	<0.01
Borrmann		
I–II	35.63 ± 21.27	
III–IV	27.72 ± 20.98	0.095
Histologic grade		
Well	36.17 ± 19.00	
Moderate	30.42 ± 22.38	
Poor	34.22 ± 21.90	NS

*NS* not significant

^a^The mean survival time, in months, was calculated by the Kaplan–Meier estimates of survival functions

^b^The *p* values were based on the log rank test. *p* < 0.05 was considered statistically significant

### Correlation Analysis Between M2-PK and VEGF Expression

There is a significant correlation between M2-PK and VEGF expression in advanced gastric cancer (*r* = 0.718, *p* < 0.01).

## Discussion

Recent studies have indicated that M2-PK and VEGF expression may be prognostic factors in colorectal cancer [15, 16]. In this study, we focused on the possible prognostic value of M2-PK and VEGF in patients with advanced gastric cancer.

Growth of tumor cells requires constant energy supply through neovascularization (angiogenesis) [17]. Tumor cells are capable of utilizing glucose for energy and metabolic substrate production even under anaerobic conditions.

VEGF, the most important regulator of the angiogenesis, promotes the recruitment and proliferation of endothelial cells and their precursors within the tumor, and thus plays a critical role in angiogenesis during tumor development [18, 19]. High VEGF expression is reported in several malignancies [20], and VEGF expression has been correlated with poor prognosis of breast cancer [21] and ovarian cancer [22]. High level of VEGF expression has been observed in gastric carcinomas [23]. Expression of VEGF has been shown to correlate positively with microvessel count and metastasis [24]. In gastric cancer, VEGF (now termed VEGF-A) is one of the strongest promoters of angiogenesis [25].

In our analysis, patients with the higher VEGF expression had significantly poorer prognosis than those with lower expression levels. The level of VEGF correlated with TNM stages of advanced gastric cancer. This result is consistent with the reported data [26]. We also observed a reduced disease-free and metastases-free survival in patients with VEGF-positive tumors. This finding is again consistent with what has previously been reported that positive VEGF is an indicator of poor survival and distant metastasis [27].

In tumor cells, increased aerobic glycolysis is one of the most common metabolic phenomenons. Tumor cells in particular express the pyruvate kinase isoenzyme type M2. The enzyme pyruvate kinase (PK) plays a central role in aerobic glycolysis, a metabolic process that is increased in tumor cells [28]. The M2-PK, which can switch between a highly active tetrameric form and an inactive dimeric form, is an important metabolic sensor to adapt tumor metabolism in nutrient and oxygen supply conditions. In tumor cells, generally the dimeric form of M2-PK is dominant and is released into the blood stream [29], and is therefore termed tumor M2-PK [30]. Tumor metastases are always characterized by homogeneous expression of large amounts of tumor M2-PK [31–33]. Elevated serum concentrations of M2-PK have been found to correlate with poor prognosis in patients with pancreaticobiliary and duodenal cancer [34]. Only limited data are available on tumor M2-PK in gastric cancer. The tumor M2-PK has been shown to be present not only in plasma, but also in feces, indicating that M2-PK may serve as a potential marker for screening colorectal and gastric cancers in high risk individuals [35, 36]. In our studies, patients with higher level of M2-PK expression had significantly poorer prognosis than those with lower M2-PK expression. The level of M2-PK correlated with tumor size (*p* = 0.0001), depth of invasion (*p* = 0.0024) and lymph node metastasis (*p* = 0.036 and *p* = 0.028, respectively). Expression level of M2-PK and VEGF in gastric cancer tissues had remarkable correlation (*r* = 0.718). Such a close correlation probably reflect the notion that in tumor tissues, M2-PK and VEGF need to operate together to provide essential environment to favor tumor growth.

Tumor size is a major determinant for patient survival. In our series, 96 (67.6 %) of patients had tumors >3 cm in size, 137 (95 %) of patients had T2–T4 tumors, 125 (88 %) of patients had lymph node metastasis, and in 77 (54 %) patients tumors were at stages III–IV. The fact that patients were generally diagnosed at advanced stages is partially due to the poor social-economic status and a lack of essential public health knowledge in the patient population. In these patients with advanced gastric cancer, M2-PK and VEGF expression, tumor size and UICC stage were all significant independent prognostic factors.

In conclusion, M2-PK and VEGF expression were positively correlated with the prognosis of advanced gastric cancer. Further studies are required to confirm the role of simultaneous analysis of these two proteins as a potential approach for determining the tumor progression and prognosis in patients with gastrointestinal malignancies.
